# First characterization of *Staphylococcus felis* in diabetic foot osteomyelitis: from intracellular persistence to phage treatment

**DOI:** 10.1128/spectrum.02340-25

**Published:** 2026-09-14

**Authors:** Cynthia N. Abi Najem, Chloé Magnan, Lucile Plumet, Vincent Jean-Pierre, Sophie Schuldiner, Albert Sotto, Jean-Philippe Lavigne, Virginie Molle

**Affiliations:** 1 Department of Microbiology and Hospital Hygiene, CHU Nîmes, VBIC, INSERM U1047, University of Montpellier 36672, Nîmes, France; 2 Department of Microbiology and Hospital Hygiene, HydroSciences Montpellier, University of Montpellier, CNRS, IRD, CHU de Nîmes 36672, Montpellier, France; 3 Department of Diabetology, Gard-Occitanie Diabetic Foot Clinic, VBIC, INSERM U1047, University of Montpellier, CHU Nîmes 36672, Nîmes, France; 4 Department of Infectious Diseases, VBIC, INSERM U1047, University of Montpellier, CHU Nîmes 36672, Nîmes, France; Houston Methodist Hospital, Houston, Texas, USA

**Keywords:** *Staphylococcus felis*, coagulase-negative staphylococci, diabetic foot osteomyelitis, intracellular persistence, zebrafish model, phage therapy, whole-genome sequencing

## Abstract

*Staphylococcus felis* is a coagulase-negative *Staphylococcus* (CoNS) primarily associated with the feline microbiota and only rarely reported in human disease. Here, we report its implication in diabetic foot osteomyelitis, and provide the first comprehensive characterization of its pathogenic potential. Two isolates (NSF001 and NSF002), recovered 5 months apart from bone biopsies of the same patient, were analyzed for growth kinetics, biofilm formation, and intracellular persistence in macrophages and osteoblasts. Both isolates proliferated efficiently, produced robust biofilm, and persisted within host cells, most markedly in osteoblasts. In a zebrafish embryo infection model, both isolates caused significant mortality, confirming their pathogenic potential *in vivo*. Whole-genome sequencing revealed conserved virulence determinants, a narrow resistome, and strain-specific genomic variations affecting genes involved in virulence regulation, phage defense, and iron acquisition. The lytic phage SAVM02, previously characterized for activity against other *Staphylococcus* species, effectively inhibited *S. felis* growth *in vitro* and conferred protection *in vivo* against lethal infection. Notably, the two sequential isolates differed in their *in vivo* virulence and phage susceptibility, paralleling these within-host microevolutionary changes and illustrating bacterial adaptation during chronic infection. Altogether, this study establishes *S. felis* as a CoNS capable of intracellular persistence, biofilm formation, and *in vivo* virulence in chronic human infection. Our findings also highlight the therapeutic potential of lytic phages against virulent CoNS species and support further investigation of phage therapy for chronic staphylococcal infections.

**IMPORTANCE:**

Coagulase-negative staphylococci (CoNS) are increasingly recognized as genuine agents of chronic infection, yet the pathogenic capacity of most individual species remains undefined. *Staphylococcus felis*, a commensal of cats only exceptionally reported in humans, had never been implicated in a chronic human infection. Here, we describe two sequential *S. felis* isolates recovered from bone biopsies of a patient with diabetic foot osteomyelitis and show that this species combines biofilm formation, intracellular persistence in macrophages and osteoblasts, and lethality in a zebrafish embryo model. Whole-genome comparison of the two isolates uncovered microevolutionary changes, most notably in iron-acquisition and genome-defense loci, that paralleled differences in virulence and phage susceptibility. These findings extend the list of CoNS capable of causing invasive human disease and provide a rationale for lytic phage therapy against emerging, difficult-to-treat staphylococcal pathogens.

## INTRODUCTION

Diabetic foot infections (DFIs) are among the most frequent and severe complications of diabetes mellitus, frequently leading to prolonged hospitalization, lower-limb amputations, and increased mortality (1, 2). These infections are typically chronic and polymicrobial, posing major challenges for both antimicrobial treatment and wound healing (3, 4). Although coagulase-negative staphylococci (CoNS) have long been considered as skin commensals or contaminants, several species are now recognized as opportunistic pathogens capable of intracellular survival, biofilm formation, and immune evasion, particularly in the context of DFIs (5–12). Among these understudied species, *Staphylococcus pettenkoferi* was the first to be functionally characterized in DFIs using complementary *in vitro* and *in vivo* models, demonstrating its capacity to persist within macrophages and keratinocytes and cause mortality in zebrafish embryos (9, 10). More recently, *S. debuckii*, another poorly characterized CoNS recovered from diabetic foot osteomyelitis (DFOM), was shown to combine intracellular persistence, host-cell cytotoxicity, and *in vivo* pathogenicity (11). Together, these studies indicate that species-level identification is progressively uncovering a broader diversity of CoNS involved in DFIs and underscore the need to functionally characterize these neglected opportunistic pathogens.

*Staphylococcus felis* is a CoNS commonly found on the skin and mucosa of healthy cats, where it is considered a commensal (13). Although occasionally associated with opportunistic infections in cats, it is exceptionally rare in humans. Animal-derived *S. felis* isolates have nonetheless been reported to display virulence-associated traits, including biofilm formation and proteolytic activity, suggesting an opportunistic pathogenic potential (14). To date, the only documented human case is that of Sips et al. (15), who reported the first confirmed cat-to-human transmission of *S. felis*, resulting in a surgical site infection; its behavior in chronic human infection has never been investigated. Here, we provide the first comprehensive functional characterization of human *S. felis* isolates, based on two sequential isolates recovered 5 months apart from deep bone biopsies of a single patient with DFOM treated at Nîmes University Hospital (France). We assessed their pathogenic potential through phenotypic characterization, intracellular persistence in macrophages and osteoblasts, virulence in a zebrafish embryo infection model, comparative whole-genome sequencing, and susceptibility to a lytic bacteriophage.

## MATERIALS AND METHODS

### Bacterial strains, culture conditions, and phage

*S. felis* isolates NSF001 and NSF002, along with *S. aureus* NSA739 (a clinical strain from our laboratory collection [16]), were isolated from patients with DFOM treated at Gard-Occitanie Diabetic Foot Clinic in Nîmes University Hospital, France. Species identification was performed using MALDI-TOF mass spectrometry (MALDI Biotyper, Bruker SAS, Wissembourg, France) and the MBT Compass Library (version 12.2.320). The two isolates yielded identification scores of 2.13 and 2.18, confirming both as *S. felis*, and were designated NSF001 and NSF002, respectively. Antibiotic susceptibility testing was assessed by disc diffusion for penicillin G, ampicillin, cefoxitin, erythromycin, clindamycin, quinupristin-dalfopristin, kanamycin, tobramycin, gentamicin, tetracycline, norfloxacin, fusidic acid, rifampicin, trimethoprim-sulfamethoxazole, and fosfomycin using Mueller-Hinton agar and antibiotic discs (Bio-Rad, Marnes-la-Coquette, France) in accordance with EUCAST guidelines (version 14.0). Inhibition zone diameters (IZDs) were measured using SIRscan Micro software (i2a, Montpellier, France), and interpreted according to EUCAST breakpoints 14.0 (https://www.eucast.org/). Minimum inhibitory concentrations (MICs) were determined by broth microdilution using Sensititre microdilution plates (FR2STENT plates for gram-positive bacteria, Thermo Fisher Scientific, Waltham, USA) for a broader panel including ampicillin, ceftaroline, ceftobiprole, clindamycin, dalbavancin, delafloxacin, levofloxacin, linezolid, rifampicin, tedizolid, vancomycin, teicoplanin, daptomycin, and trimethoprim-sulfamethoxazole. Interpretation of results was based on *Staphylococcus* spp. breakpoints defined in EUCAST 14.0. When genus-level breakpoints were unavailable, *S. aureus* or CoNS-specific thresholds were applied. For fosfomycin and kanamycin, older EUCAST versions (11.0 and 13.0) were used due to the absence of updated disc diffusion criteria.

Staphylococcal isolates were routinely cultured on tryptic soy agar (TSA) or in tryptic soy broth (TSB) at 37°C with shaking at 180 rpm. Bacterial growth was monitored over 24 hs by measuring optical density at 600 nm (OD_600_) using a Spark microplate reader (Tecan, Grödig, Austria), under continuous shaking at 108 rpm. The lytic phage SAVM02 used in this study was previously isolated and characterized by Plumet et al. (17).

### Biofilm formation assay

Biofilm production was evaluated using the Congo red broth (CRB) assay. CRB was prepared by supplementing TSB with 3.6% (wt/vol) sucrose before autoclaving; filter-sterilized Congo red was then added to a final concentration of 0.08% (wt/vol). Overnight cultures of *S. aureus* NSA739 and *S. felis* NSF001 and NSF002 were diluted to an initial OD_600_ of 0.2 in 10 mL of CRB and incubated statically at 37°C in 50 mL Falcon tubes. CRB-only tubes served as blanks. Three biological replicates per strain were analyzed at each time point (0, 4, 6, and 24 h). At each time point, cultures were centrifuged at 4,000 rpm for 5 min. The supernatants were transferred to 96-well plates for absorbance measurement at 490 nm (OD_490_) to quantify residual Congo red. Pellets were resuspended in phosphate-buffered saline (PBS), and OD_600_ was recorded to normalize for bacterial growth. Congo red retention, reflecting biofilm matrix production, was calculated as the difference between the OD_490_ of the blank and the sample supernatant, adjusted using a standard curve of known dye concentrations, and normalized to OD_600_.

### Infection assays in macrophages and osteoblasts

The murine macrophage cell line RAW 264.7 (ATCC TIB-71) and the human osteosarcoma-derived osteoblast-like cell line MG63 were maintained in Dulbecco’s modified Eagle medium (DMEM, Thermo Fisher Scientific) supplemented with 10% fetal calf serum (Thermo Fisher Scientific). MG63 cells were additionally supplemented with 1% l-glutamine and 0.1% penicillin/streptomycin (Gibco). Cultures were maintained at 37°C in a humidified 5% CO_2_ atmosphere. Bacterial strains were grown in TSB to mid-exponential phase (OD_600_ = 0.6–0.8), harvested by centrifugation (5 min, 4,000 rpm), and resuspended in sterile PBS. RAW 264.7 macrophages (5 × 10⁵ cells/mL) and MG63 osteoblasts (2 × 10^5^ cells/mL) were seeded in 24-well plates and infected at a multiplicity of infection (MOI) of 20:1 (macrophages) or 100:1 (osteoblasts) for 1 h at 37°C, 5% CO_2_. After infection, extracellular bacteria were eliminated by a 30-min incubation with gentamicin (100 µg/mL), followed by incubation for 5 and 24 h in fresh medium containing lysostaphin (5 µg/mL) to prevent extracellular regrowth. At each time point (0, 5, and 24 h), infected cells were lysed with 0.1% Triton X-100 in PBS. Serial dilutions of cell lysates were plated on TSA and incubated at 37°C for 24 h to enumerate intracellular bacteria (CFU).

### Cell viability assay

Cytotoxicity was evaluated by quantifying the release of lactate dehydrogenase (LDH) into the culture supernatant using the LDH Cytotoxicity Assay Kit (MedChemExpress, USA). RAW 264.7 macrophages and MG63 osteoblasts were infected with *S. felis* NSF001, NSF002, or *S. aureus* NSA739 at MOIs of 20:1 and 100:1, respectively. To simulate total cytotoxic impact, extracellular bacteria were not removed. LDH release was quantified on culture supernatant using a Spark microplate reader (Tecan, Grödig, Austria) by measuring absorbance at 490 nm.

### Phage susceptibility assay

The lytic activity of phage SAVM02 against *S. felis* isolates was evaluated using a phage liquid culture assay in 96-well plates. Bacterial suspensions were adjusted to 3 × 10⁷ CFU/mL and incubated with SAVM02 at MOIs of 0.01, 0.1, and 1, corresponding to final phage concentrations ranging from 3 × 10⁵ to 3 × 10⁷ PFU/mL. Cultures were maintained at 37°C with continuous shaking at 108 rpm, and bacterial growth was monitored over 16 h by measuring OD_600_ using a Spark microplate reader.

### Zebrafish embryo infections and phage treatment

Zebrafish (*Danio rerio*) embryos from the AB wild-type line were used prior to the onset of exogenous feeding (i.e., before 120 h post-fertilization [hpf]), and thus were exempt from animal experimentation regulations. All procedures complied with European Union guidelines for the care and use of laboratory animals (Directive 2010/63/EU). Embryos were obtained by natural spawning and maintained under standard laboratory conditions. Overnight cultures of *S. felis* NSF001, *S. felis* NSF002, and *S. aureus* NSA739 were grown in TSB to mid-exponential phase (OD_600_ = 0.7–0.9), centrifuged, washed twice with PBS, and resuspended in PBS containing 10% glycerol. Aliquots were stored at −80°C and plated after thawing to determine bacterial concentrations. On the day of injection, bacterial suspensions were adjusted in PBS with 0.1% phenol red to a final concentration of 10,000 CFU/nL. At 30 hpf, embryos were dechorionated, anesthetized with Tricaine, and injected with 1 nL of bacterial suspension into the hindbrain ventricle. Phage suspensions were prepared from high-titer SAVM02 stocks diluted in PBS containing 0.1% phenol red and 60 μM fluorescent Dextran to reach final concentrations of 5 × 10⁸ PFU/mL. One hour post-infection, embryos received a second microinjection of 1 nL of the phage solution into the hindbrain ventricle; PBS alone served as a control. Each embryo was individually injected and transferred to a separate well of a 96-well plate. To verify the injected dose, five embryos per condition were euthanized immediately after injection using Tricaine and individually homogenized in 200 µL of PBS containing 0.1% Triton X-100. Homogenates were serially diluted and plated on TSA for CFU enumeration. For phage titration, the same homogenates were diluted and plated using a soft agar overlay containing the SAVM02 production strain.

### Whole-genome sequencing and analysis

Whole-genome sequencing was performed by the Micro&Bio platform (Department of Microbiology and Hospital Hygiene, Nîmes University Hospital). Genomic DNA was extracted using the DNeasy UltraClean Microbial kit (Qiagen, Courtaboeuf, France) and eluted in 50 µL. DNA concentrations were quantified using a Qubit 4 fluorometer and the Qubit dsDNA High-Sensitivity assay (Thermo Fisher Scientific, Villebon-sur-Yvette, France). Whole-genome libraries were prepared with the DNA Prep kit (Illumina, San Diego, USA) and sequenced on a MiSeq instrument (2 × 250 bp, 39-h run). Read quality was assessed using FastQC v0.12.1. Draft genomes of *S. felis* NSF001, *S. felis* NSF002, and *S. aureus* NSA739 were assembled *de novo* using Unicycler (version 0.5.0). Average nucleotide identity (ANI) was calculated with OAT v0.93.10, and digital DNA–DNA hybridization (dDDH) values were estimated using the Genome-to-Genome Distance Calculator 3.0 (https://ggdc.dsmz.de/). Confirmation of species corresponded to ANI ≥ 95–96% and dDDH ≥ 70% vs *S. felis* ATCC 49168. Genome annotations were generated using DFAST (https://dfast.ddbj.nig.ac.jp/) and Bakta (Galaxy implementation v1.9.4). Virulence- and antimicrobial resistance-associated genes were identified by combining the results of ResFinder v4.7.2 and VirulenceFinder v3.2.0 (Center for Genomic Epidemiology) with the annotated gene clusters generated by Roary v3.13.0 (Galaxy). Core-genome single-nucleotide polymorphisms (SNPs) were detected using Snippy v4.6.0 (Galaxy) on Bakta-annotated assemblies. CRISPR-Cas loci were identified using CRISPRCasFinder v1.1.0, and prophage regions were detected with PHASTEST v1.1.0 via the Proksee platform (https://proksee.ca). Missing genomic segments were resolved by BLAST analysis using the CGView server integrated in Proksee.

### Statistical analyses

GraphPad Prism version 11.0.2 was used to perform statistical analyses, which are indicated in the relevant figure legends.

## RESULTS

### Case report

*S. felis* isolates NSF001 and NSF002 were isolated from a 75-year-old man with DFOM treated at the Gard-Occitanie Diabetic Foot Clinic, Nîmes University Hospital, France. NSF001 and NSF002 were recovered from deep bone biopsies obtained during two surgical debridements with necrosectomies performed in January and June 2024, respectively. Following the first procedure, the patient received a 6-week course of piperacillin–tazobactam and linezolid. Bone biopsies yielding NSF001 were associated with polymicrobial cultures composed predominantly of gram-positive commensals and anaerobic bacteria, whereas biopsies yielding NSF002 harbored a more diverse polymicrobial community including Enterobacterales, *Enterococcus* spp., *Corynebacterium* spp., and anaerobic bacteria, consistent with the complex microbial ecosystem of chronic DFOM. The subsequent clinical course was marked by delayed wound healing and recurrent local necrosis, ultimately requiring an additional forefoot surgical procedure in October 2024. Intraoperative bone cultures obtained during this intervention no longer yielded *S. felis* but remained polymicrobial. Following prolonged local wound care and antimicrobial therapy, the patient gradually improved, and complete wound healing was achieved in March 2025, avoiding major lower-limb amputation.

### Growth kinetics and biofilm formation of *S. felis* isolates

**Fig 1:**
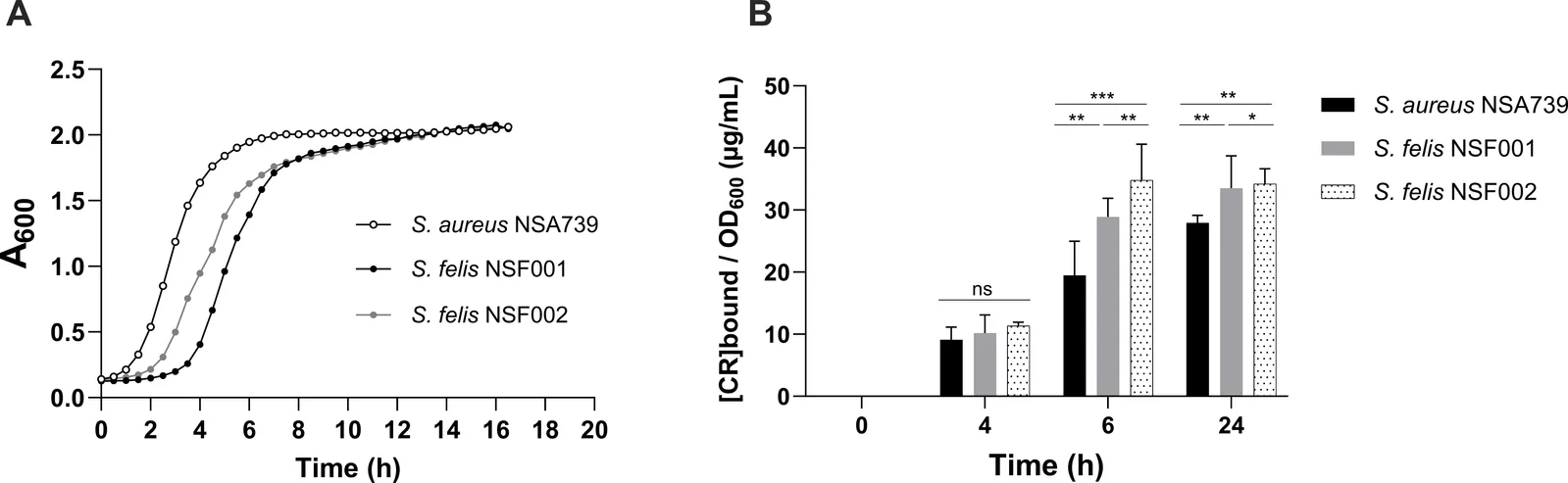
Planktonic growth and biofilm formation of *S. felis* isolates. (**A**) Growth kinetics of *S. aureus* NSA739 (open circles), *S. felis* NSF001 (black circles), and NSF002 (gray circles) in tryptic soy broth (TSB) at 37°C under agitation (108 rpm) over 24 h. Curves represent the mean optical density at 600 nm (OD_600_) values from three independent biological replicates. (**B**) Quantification of biofilm formation using the Congo red broth assay at 4, 6, and 24 h post-inoculation. Results are expressed as retained Congo red (CR) signal normalized to planktonic bacterial density (OD_600_). Statistical significance was determined using two-way ANOVA followed by Tukey’s multiple comparisons test (*P < 0.05, **P < 0.01, ***P < 0.001, and ns: not significant).

To gain initial insights into the biological behavior of the two *S*. *felis* isolates recovered from DFOM, we first assessed their growth dynamics and biofilm-forming capacity, two phenotypic traits known to influence persistence in chronic infections. Growth curves in TSB medium showed that all three strains reached similar optical densities after 24 h. However, differences were observed in the onset of exponential growth: *S. aureus* NSA739 entered the exponential phase most rapidly, followed by *S. felis* NSF002, while *S. felis* NSF001 exhibited a delayed onset (Fig. 1A). Biofilm-associated matrix production was assessed with the CRB assay, in which dye retention reflects the amount of extracellular matrix produced. No significant difference in Congo red retention was observed among strains at 4 h, but from 6 h onward both *S. felis* isolates retained more dye than *S. aureus* NSA739, with NSF002 giving the strongest signal. By 24 h, all three strains showed substantial dye retention, slightly higher for NSF001 and NSF002 than for NSA739 (Fig. 1B). These findings show that, despite slower initial growth, both *S. felis* isolates are efficient matrix producers after 24 h, a trait that may favor persistence in the chronic wound environment.

### Intracellular survival of *S. felis* isolates in macrophages

**Fig 2:**
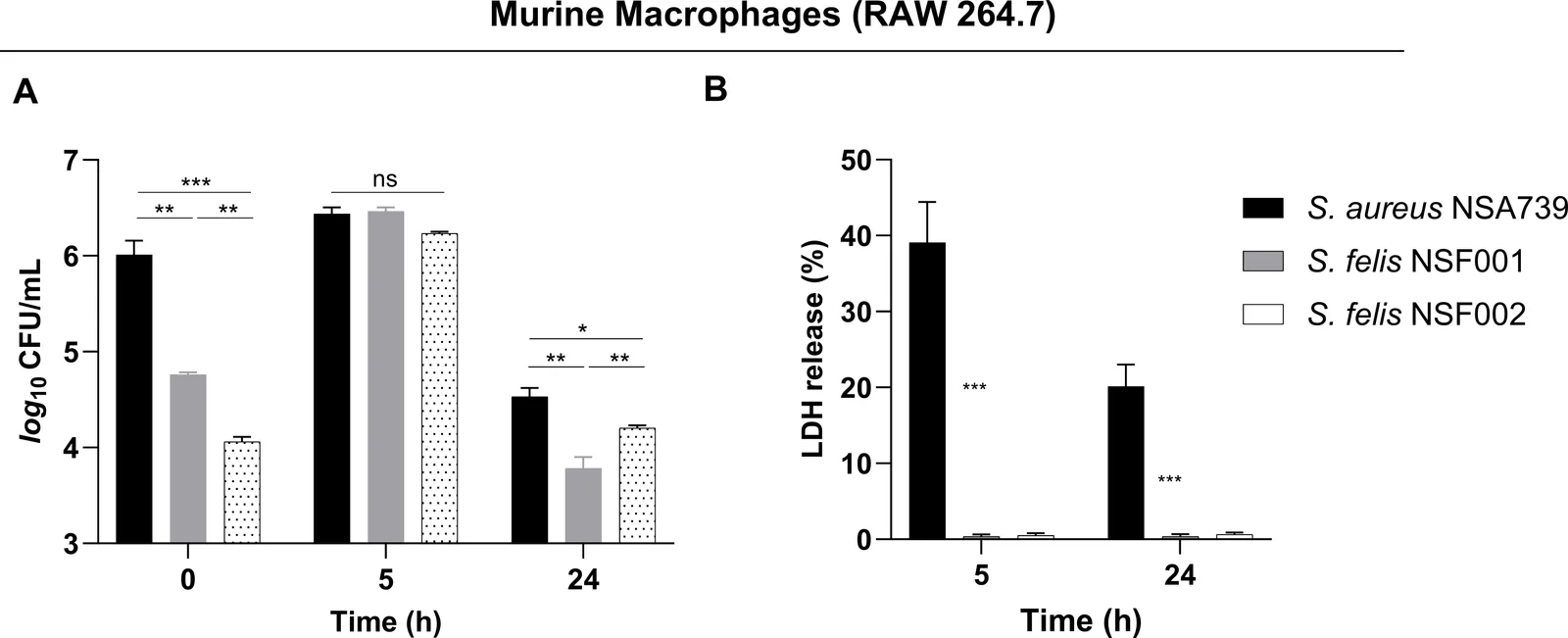
Intracellular survival and cytotoxicity of *S. felis* isolates in murine macrophages. RAW 264.7 macrophages were infected with *S. aureus* NSA739, *S. felis* NSF001, or *S. felis* NSF002 at a multiplicity of infection (MOI) of 20 for 1 h at 37°C. After infection, extracellular bacteria were removed by gentamicin treatment (100 µg/mL, 30 min), followed by incubation in complete medium containing lysostaphin (5 µg/mL) to prevent overgrowth of extracellular bacteria potentially released from lysed cells. (**A**) Intracellular bacterial load was quantified at 0, 5, and 24 h post-antibiotic treatment. CFUs were enumerated from lysed macrophages. (**B**) Cytotoxicity was assessed by measuring LDH release at 5 and 24 h post-infection using the MCE LDH Cytotoxicity Assay Kit. Data represent the mean ± SD from five independent experiments. Statistical significance was determined by two-way ANOVA followed by Tukey’s multiple comparisons test (*P < 0.05, **P < 0.01, ***P < 0.001, and ns: not significant).

Intracellular persistence is a well-recognized immune-evasion strategy that may contribute to chronicity, particularly in DFIs. To explore this potential, we quantified the intracellular survival of NSF001 and NSF002 in RAW 264.7 murine macrophages relative to *S. aureus* NSA739. Cells were infected at an MOI of 20, and intracellular bacterial loads were measured at 0, 5, and 24 h post-infection after elimination of extracellular bacteria. Both *S. felis* isolates showed significantly lower initial uptake than *S. aureus* NSA739, with NSF002 displaying the lowest T0 count (Fig. 2A). Intracellular loads of both isolates declined over time, whereas *S. aureus* maintained higher intracellular counts at 24 h, indicating greater persistence. Although NSF001 and NSF002 reached similar levels at 5 h, NSF001 declined more sharply between 5 and 24 h, suggesting subtle differences in their intracellular fitness. Consistent with a non-cytotoxic intracellular lifestyle, LDH release from *S. felis*-infected macrophages was minimal and stable over time, whereas *S. aureus* induced substantial LDH release at 5 h that decreased but remained elevated at 24 h (Fig. 2B). Thus, both *S. felis* isolates transiently persisted within macrophages while inducing limited cytotoxicity, a phenotype compatible with persistence during chronic infection.

### Invasion and intracellular persistence in osteoblasts

**Fig 3:**
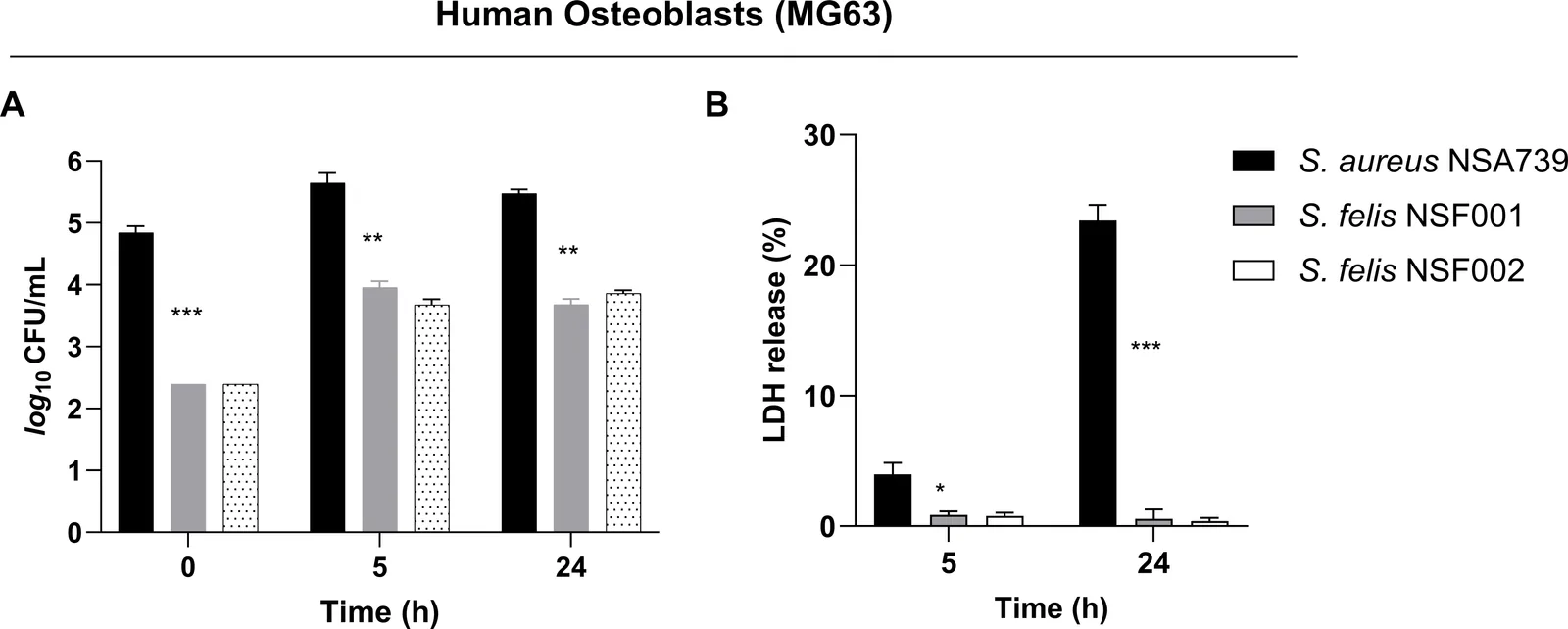
Intracellular survival and cytotoxicity of *S. felis* isolates in human osteoblasts. MG63 osteoblasts were infected with *S. aureus* NSA739 or *S. felis* isolates (NSF001 and NSF002) at a multiplicity of infection (MOI) of 100 for 1 h at 37°C. After infection, extracellular bacteria were removed by gentamicin treatment (100 µg/mL, 30 min), followed by incubation in complete medium containing lysostaphin (5 µg/mL) to prevent overgrowth of extracellular bacteria potentially released from lysed cells. (**A**) Intracellular CFUs were quantified at 0, 5, and 24 h post-treatment. (**B**) LDH release was measured at 5 and 24 h post-infection using the MCE LDH Cytotoxicity Assay Kit (cells seeded in 96-well plates; extracellular bacteria were not removed). Data represent the mean ± SD from five independent experiments. Statistical significance was determined using two-way ANOVA followed by Tukey’s multiple comparisons test (*P < 0.05, **P < 0.01, ***P < 0.001).

Given the role of osteoblasts as potential intracellular reservoirs in DFOM, we next evaluated the ability of *S. felis* isolates to invade and persist within human MG63 osteoblasts. As shown in Fig. 3A, both *S. felis* isolates exhibited significantly lower internalization at the initial time point (T0) compared to *S. aureus* NSA739, suggesting reduced initial uptake by osteoblasts. However, while NSA739 maintained stable intracellular levels over time, both *S. felis* isolates showed an increase in intracellular CFUs between 0 and 5 h, which remained stable up to 24 h. No significant differences were observed between NSF001 and NSF002. To evaluate the cytotoxic impact of intracellular persistence, LDH release was measured at 5 and 24 h post-infection (Fig. 3B). Infection with *S. aureus* NSA739 induced significant LDH release at both time points, reflecting high cytotoxicity. In contrast, osteoblasts infected with either *S. felis* isolate exhibited minimal LDH release, suggesting limited cytotoxic effects despite bacterial persistence. Together, these findings indicate that both isolates persisted within osteoblasts while inducing limited cytotoxicity, a characteristic that may favor bacterial persistence in bone tissue.

### Virulence of *S. felis* isolates in the zebrafish model

**Fig 4:**
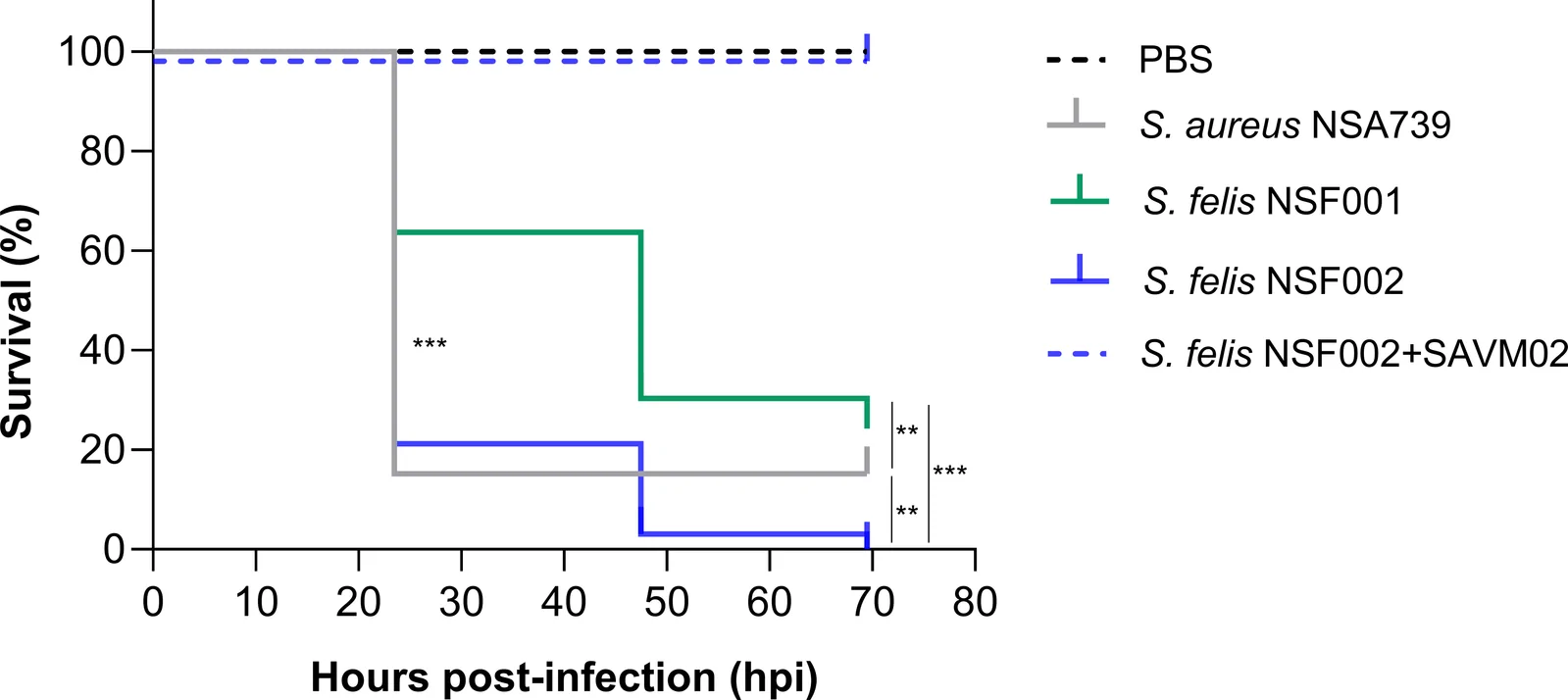
Zebrafish embryo survival following infection with *S. felis* isolates and phage treatment. Zebrafish embryos at 30 h post-fertilization were microinjected into the hindbrain ventricle with ~10^4^ CFU/embryo of *S. aureus* NSA739, *S. felis* NSF001, or NSF002. For the phage treatment condition, embryos infected with NSF002 received an additional injection of phage SAVM02 (500 PFU/embryo) at one hour post-infection (hpi). Embryo survival was monitored over 70 h. Data represent the mean of five independent experiments, each performed with 30 embryos. Statistical significance was determined by the Log-rank (Mantel–Cox) test (**, *P* < 0.01; ***, *P* < 0.001).

To assess the *in vivo* pathogenic potential of *S. felis* isolates, we used the zebrafish embryo infection model, previously established in our laboratory to evaluate the virulence of various *Staphylococcus* species. Embryos were microinjected with ~10^4^ CFU of *S. aureus* NSA739 or *S. felis* NSF001 and NSF002, and survival was monitored over a 70-h period (Fig. 4). *S. aureus* NSA739 induced rapid and pronounced mortality, with survival dropping to ~20% within the first 24 h and remaining stable thereafter. A similar early lethality pattern was observed with *S. felis* NSF002, which continued to cause progressive mortality, resulting in near-complete embryo death by 70 h post-infection. In contrast, *S. felis* NSF001 exhibited a delayed virulence profile, with ~60% of embryos surviving at 24 h, followed by a gradual decline to ~30% at 70 h. These findings show that both *S. felis* isolates induced significant mortality in the zebrafish embryo model, supporting their pathogenic potential *in vivo*.

### Genomic features and comparative analysis of *S. felis* isolates

**Fig 5:**
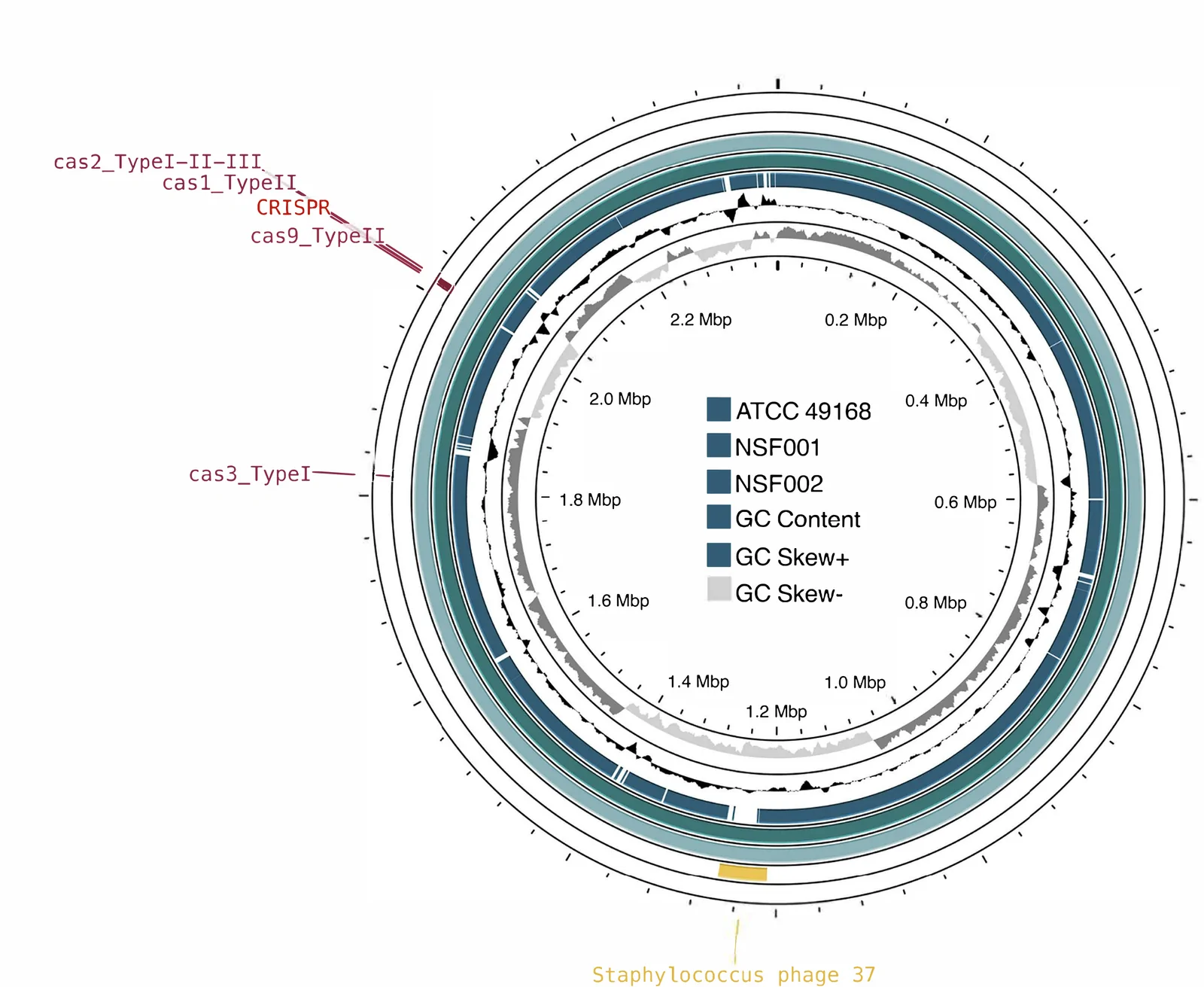
Comparative genome analysis of the *S. felis* isolates and reference strain. Circular genome maps of *S. felis* ATCC 49168 (dark blue), NSF001 (dark green), and NSF002 (light green) generated using the CGView server via PROKSEE. Genome regions absent in ATCC 49168 but present in clinical isolates are depicted as gaps. Shared prophage and CRISPR-Cas elements are annotated.

Whole-genome sequencing of *S. felis* NSF001 and NSF002 was performed and compared to the reference strain ATCC 49168 (Fig. 5). Genome sizes were 2,396,232 bp for NSF001, 2,396,476 bp for NSF002, and 2,479,423 bp for ATCC 49168 (Table 1). Both clinical isolates exhibited a GC content of 34.8%, slightly lower than the 35.2% observed in the reference strain. Comparative genomic analysis revealed high similarity, with ANI values of 99% and dDDH values of 90.9% for NSF001 and 90.8% for NSF002, confirming species-level identity. Despite this overall similarity, NSF001 and NSF002 harbored a set of unique genes absent from ATCC 49168.

**TABLE 1:** Genome characteristics of NSF001, NSF002, ATCC 49168, and NSA739 isolates^*a*^

Characteristic	ATCC 49168	NSF001	NSF002	NSA739
Biosample	SAMN08712993	SAMN57405566	SAMN57405567	SAMN57405617
Bacterial species	*Staphylococcus felis*	*Staphylococcus felis*	*Staphylococcus felis*	*Staphylococcus aureus*
Genome size (bp)	2,479,423	2,396,232	2,396,476	2,794,675
GC content (%)	35.2	34.8	34.8	32.7
Number of CDSs	2,339	2,270	2,261	2,635
Locality	Japan	Nîmes (France)	Nîmes (France)	Nîmes (France)

^*a*^ CDS, coding DNA sequences.

Antibiotic susceptibility testing revealed that both isolates were fully susceptible to all tested agents, including (lipo)glycopeptides, with MICs of 0.5 mg/L for vancomycin, <0.25 mg/L for teicoplanin, and <0.03 mg/L for dalbavancin, as well as to advanced-generation cephalosporins, with MICs of 0.5 mg/L for ceftaroline and ceftobiprole. Because species-specific breakpoints are not available for *S. felis*, susceptibility was interpreted using *S. aureus*/CoNS criteria. In line with these phenotypic findings, resistome analysis (Table S1) revealed a limited repertoire of resistance genes. Both genomes encoded a predicted ABC-F family ribosomal protection protein, typically associated with low-level macrolide resistance, and a major facilitator superfamily (MFS) transporter implicated in multidrug efflux. These genetic determinants are commonly found in staphylococci and are not necessarily associated with clinically relevant resistance. Their presence is therefore consistent with the observed full susceptibility profile, suggesting that these elements have minimal impact on antibiotic efficacy in these strains.

Virulence gene profiling (Table S2) revealed that NSF001 and NSF002 shared a broad set of conserved determinants also present in *S. aureus* NSA739, including regulatory systems (*agr*, *sarA*, *mgrA*, *vraSR*, *saeSR*, and *lytSR*), stress response genes (*katA*, *tuf*, *eno*, and *cspA*), and immune evasion factors such as *sbi* and *lip2/geh*. Notably, all *S. felis* isolates harbored a gene encoding a trypsin-like serine peptidase. The predicted protein shared 67.1% amino acid identity over 100% query coverage with *S. aureus* exfoliative toxin D by BLASTp and was absent from *S. aureus* NSA739. Conversely, canonical cytotoxins such as *hlgABC*, *sea*, *selX*, *lukDE*, and *aur* were exclusive to *S. aureus*, highlighting its broader cytotoxic arsenal. Regarding capsular biosynthesis, only *cap8O* was conserved in *S. felis*, whereas NSA739 carried the full *cap8* operon, potentially conferring enhanced immune evasion.

In terms of biofilm-associated genes (Table S3), *S. felis* isolates exhibited a more limited repertoire compared to *S. aureus*. While some conserved elements were present, such as *fnbpB*, *ebpS*, and regulators like *agrADBC* and *sigB,* key adhesion (*sasF*, *sasA*, *eap*, and *emp*) and matrix accumulation genes (*icaADBC*, *sasG*, and *sbp*) were absent. Similarly, serine proteases involved in biofilm structuring (*splB*, *splC*, *splD*, and *splF*) were only detected in *S. aureus*, whereas *S. felis* retained only *splA*. These differences suggest that *S. felis* may rely on alternative or simplified mechanisms for biofilm formation, potentially reflecting adaptation to different ecological niches or host environments.

Finally, 24 SNPs were identified between NSF002 and NSF001, including 22 within coding sequences (Table S4). Several of these variants affected genes known to modulate bacterial virulence, adaptation, and genome defense. Notably, two distinct mutations were found in *walK* (encoding the sensor histidine kinase of the WalKR two-component system that governs cell wall metabolism), and in *stp1/ireP* (encoding a PP2C-type Ser/Thr phosphatase implicated in stress response and pathogenicity control). Additional mutations were identified in genes encoding a type I restriction–modification system, potentially influencing protection against foreign DNA and phage infection. Other variants affected a protein A-like gene (potentially associated with immune evasion), an HNH-type endonuclease (involved in DNA protection), and an ABC-type Fe^3+^ transporter. Further mutations were observed in regulatory and metabolic genes such as *argR*, as well as in several hypothetical proteins, underscoring the genomic divergence between the two clinical isolates.

A more detailed comparison between NSF001 and NSF002 revealed the presence of one gene exclusive to NSF002, namely *feoA*, encoding a cytoplasmic partner of the FeoB iron transporter. Deletion of *feoA* has been shown to impair iron uptake, a process essential for virulence regulation in *Staphylococcus* species (18, 19). This unique genomic feature may contribute to the enhanced virulence observed in NSF002. In addition to these gene-level characteristics, both *S. felis* NSF001 and NSF002 genomes shared a conserved *Staphylococcus* phage 37 prophage and an identical CRISPR–Cas architecture, comprising a syntenic type II system (*cas1*, *cas2*, and *cas9*) and a solitary *cas3* gene, indicative of a degenerate type I system (Fig. 5). These elements may play roles in genome stability, phage resistance, and horizontal gene transfer, further shaping the pathogenic potential of these *S. felis* strains.

### Phage susceptibility of *S. felis* isolates and therapeutic efficacy in the zebrafish model

**Fig 6:**
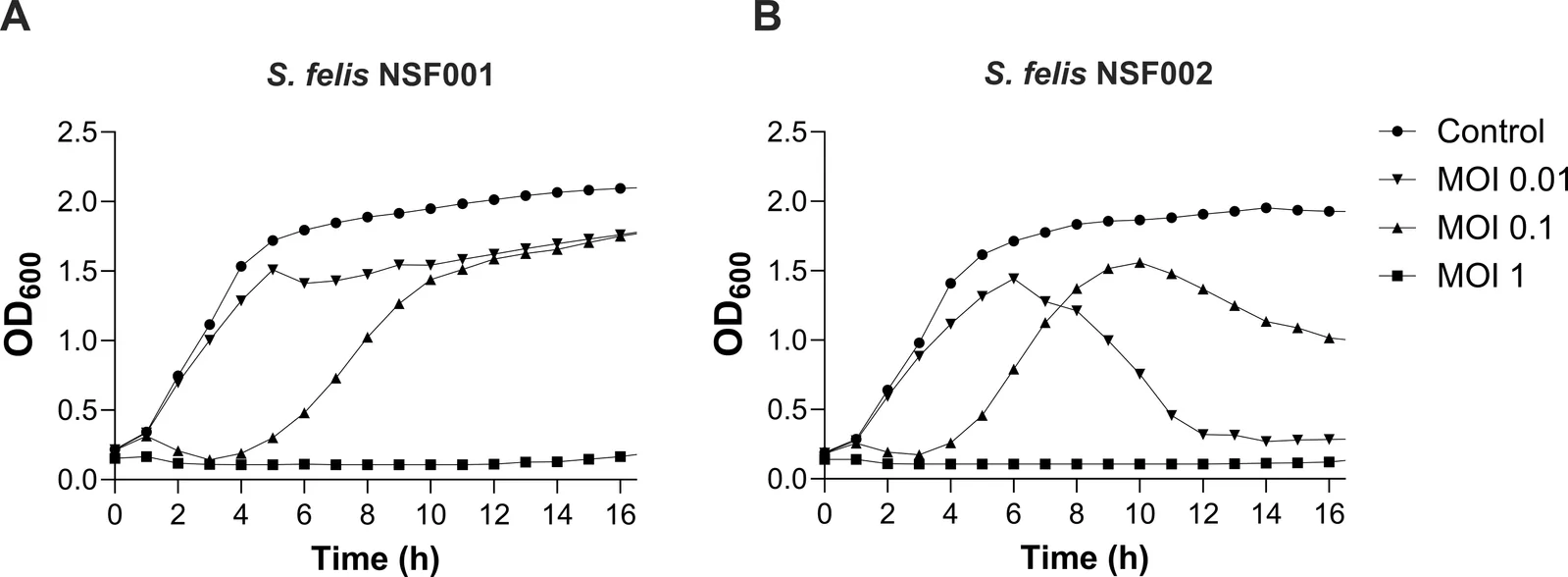
Inhibition of *S. felis* growth by phage SAVM02 in planktonic culture. Growth kinetics of *S. felis* isolates NSF001 (**A**) and NSF002 (**B**) were monitored in the presence of phage SAVM02 at multiplicities of infection (MOIs) of 0.01, 0.1, and 1. Bacterial cultures were inoculated in 96-well plates at 37°C with continuous shaking, and OD_600_ was recorded hourly over 16 h. Data shown are representative of three independent experiments performed in triplicate.

Given the pathogenic potential of both *S. felis* isolates, we next evaluated the activity of the lytic phage SAVM02, previously characterized for its broad activity against *Staphylococcus* species (17), first against both isolates *in vitro* and subsequently *in vivo*. Phage activity was assessed by monitoring bacterial growth in planktonic culture at increasing MOIs (Fig. 6). For NSF001 (Fig. 6A), an MOI of 1 completely inhibited bacterial growth over 16 h, whereas lower MOIs (0.1 and 0.01) delayed growth in a dose-dependent manner before regrowth occurred. In contrast, NSF002 (Fig. 6B) exhibited greater susceptibility to SAVM02. Although an MOI of 1 also completely inhibited bacterial growth, lower MOIs resulted in only transient bacterial proliferation followed by a progressive decline in OD_600_, consistent with sustained phage-mediated lysis. Based on both its higher virulence in the zebrafish model and its greater *in vitro* susceptibility to SAVM02, NSF002 was selected for *in vivo* phage therapy evaluation. Embryos infected with NSF002 and subsequently treated with SAVM02 exhibited survival rates comparable to those of uninfected controls (Fig. 4). Together, these findings demonstrate the robust activity of SAVM02 against *S. felis* while highlighting isolate-dependent differences in phage susceptibility.

## DISCUSSION

To our knowledge, this is the first report implicating *S. felis* in a chronic human infection, based on two isolates recovered 5 months apart from deep bone biopsies in a case of DFOM. Traditionally considered as a feline commensal, *S. felis* has rarely been associated with human pathology. Its repeated isolation from the same patient, in conjunction with clinical signs of chronic infection, strongly supports its role as an opportunistic pathogen. These findings contribute to the growing recognition of CoNS as underestimated contributors to chronic infections, particularly in immunocompromised individuals or polymicrobial environments (20). Although no information was available regarding the patient’s contact with cats, the ecological niche of *S. felis* suggests that zoonotic transmission remains a plausible hypothesis, warranting further investigation through exposure history and strain tracking in future studies. From a diagnostic standpoint, the rarity of *S. felis* in human specimens means that it may be overlooked or misidentified by routine laboratory workflows, particularly where identification databases do not include this animal-associated species. Its recovery here relied on MALDI-TOF mass spectrometry and was confirmed genomically by ANI and dDDH against the type strain, underscoring the value of up-to-date spectral databases and, where needed, whole-genome sequencing for the accurate identification of rare CoNS. Broader adoption of such tools may reveal that the clinical burden of *S. felis* and of other under-recognized CoNS is currently underestimated.

Phenotypic characterization of NSF001 and NSF002 revealed traits consistent with persistence, including efficient planktonic growth and robust biofilm formation. Although *S. felis* carried a more limited repertoire of adhesion and matrix maturation genes than *S. aureus* and lacked the *icaADBC* operon, both isolates produced substantial biofilm, pointing to *ica*-independent matrix components and alternative or simplified biofilm strategies. This observation aligns with recent findings in other CoNS species, such as *S. debuckii* and *S. pettenkoferi*, which also form robust biofilms despite lacking classical biofilm-associated genes (9–11). These convergent phenotypes suggest that biofilm formation in CoNS may rely on distinct evolutionary pathways adapted to specific host niches.

Intracellular survival assays revealed distinct behaviors in macrophages and osteoblasts. While *S. felis* isolates survived only transiently within macrophages, with intracellular bacterial loads progressively declining over time, they persisted more efficiently within osteoblasts, where intracellular CFUs increased before stabilizing. Importantly, neither cell type exhibited substantial cytotoxicity following *S. felis* infection. This non-cytotoxic intracellular lifestyle is consistent with previous reports on CoNS and supports the role of *S. felis* in chronic infections, particularly in contexts where immune clearance and antibiotic penetration are compromised (9, 11, 21). Together with their robust biofilm-forming capacity, these findings suggest that *S. felis* can colonize protected intracellular niches, thereby promoting persistence and recurrence. Notably, despite the full *in vitro* susceptibility of both isolates to all antibiotics tested, including those administered to the patient, infection recurred 5 months later, suggesting that protected intracellular persistence, rather than acquired antibiotic resistance, could account for treatment failure in this case.

Although both isolates exhibited similar *in vitro* phenotypes, NSF002 induced significantly higher mortality in the zebrafish model, suggesting increased virulence *in vivo*. This difference is unlikely to be explained by a broader toxin repertoire, as both isolates shared similar profiles of exotoxins and proteases. Comparative genomic analysis revealed a highly conserved virulome between NSF001 and NSF002, with no additional classical virulence factors identified in NSF002. However, NSF002 uniquely harbored the *feoA* gene, encoding a cytoplasmic partner of the FeoB ferrous iron transporter, which contributes to iron acquisition under the iron-restricted conditions imposed by the host, a process linked to staphylococcal fitness and virulence, notably in *Staphylococcus lugdunensis* (22). The acquisition or retention of *feoA* in NSF002 may therefore be associated with its higher *in vivo* virulence, although this link remains correlative and awaits functional confirmation. These observations highlight how microevolutionary changes between sequential isolates from the same patient can influence host-pathogen interactions and clinical outcomes. Nevertheless, differential gene expression, regulatory shifts, or host-induced transcriptomic responses may also play critical roles, even in the absence of major genomic differences. The small number of core-genome variants (24 SNPs) is more consistent with a single lineage micro-evolving within the host than with co-infection by two independent clones, supporting an interpretation of within-host adaptation.

The therapeutic potential of the lytic phage SAVM02, originally selected against *S. aureus*, was also investigated. Despite the lack of pre-existing data on phage therapy targeting *S. felis*, SAVM02 demonstrated potent lytic activity *in vitro* and conferred significant protection in the zebrafish model, particularly against the more virulent NSF002 isolate. Interestingly, NSF002, which harbored mutations in genes associated with restriction-modification systems, appeared more susceptible to phage killing. This observation suggests that genomic variation in bacterial defense systems may modulate phage sensitivity, echoing findings in *S. aureus* where altered R-M systems have been linked to enhanced phage transduction and therapeutic efficacy (23, 24). Moreover, the increased susceptibility of NSF002 to SAVM02 may also relate to the identified mutation in *walK*, which encodes the sensor histidine kinase of the WalKR two-component system central to cell wall metabolism. Alterations in WalKR modify cell-wall architecture and surface charge, potentially affecting phage adsorption and penetration (25).

This study has several limitations. First, it is based on a single patient, precluding broader epidemiological conclusions or generalization of the findings. In addition, the absence of documented cat exposure prevents any conclusion regarding the route of acquisition. Second, virulence was assessed using zebrafish embryos and cell culture models, which, although well-established, cannot fully recapitulate the complexity of human DFOM. Finally, the genotype-phenotype associations proposed here, particularly those involving *feoA*, *walK*, and restriction-modification system genes, remain correlative. Further functional studies, including targeted mutagenesis and transcriptomic analyses, will be required to establish causal relationships.

In conclusion, our study provides the first comprehensive characterization of *S. felis* associated with chronic human infection, identifying this previously unrecognized CoNS species as capable of intracellular persistence, biofilm formation, and lethal infection in an *in vivo* zebrafish embryo model. Comparative genomic analysis of sequential isolates revealed adaptive changes likely driven by selective pressures during infection, favoring traits that enhance persistence and pathogenicity. These findings underscore the importance of genomic surveillance, even for rare CoNS species, in uncovering adaptive trajectories and novel determinants of chronic infection. Finally, our results highlight the promise of lytic phages as targeted therapeutics against staphylococcal pathogens and support further investigation into phage-bacterium-host interactions in the context of DFOM.

## Data Availability

The genome assemblies of *S. felis* NSF001, NSF002, and *S. aureus* NSA739 have been deposited in GenBank under BioProject PRJNA1456407 (accession numbers JBXQSI000000000, JBXQSH000000000, and JBXQQJ000000000; BioSamples SAMN57405566, SAMN57405567, and SAMN57405617). The data sets generated or analyzed during this study are available from the corresponding author upon reasonable request.

## SUPPLEMENTAL MATERIAL

Tables S1 to S4 spectrum.02340-25-s0001.pdf Comparative analysis of antimicrobial resistance, virulence and biofilm-associated genes, and genomic variants among the studied *Staphylococcus* strains.

ASM does not own the copyrights to Supplemental Material that may be linked to, or accessed through, an article. The authors have granted ASM a non-exclusive, world-wide license to publish the Supplemental Material files. Please contact the corresponding author directly for reuse.

## References

[1] Singh N, Armstrong DG, Lipsky BA. 2005. Preventing foot ulcers in patients with diabetes. JAMA 293:217–228. doi:10.1001/jama.293.2.217 15644549

[2] Lipsky BA, Senneville É, Abbas ZG, Aragón-Sánchez J, Diggle M, Embil JM, Kono S, Lavery LA, Malone M, van Asten SA, Urbančič-Rovan V, Peters EJG, International Working Group on the Diabetic Foot (IWGDF). 2020. Guidelines on the diagnosis and treatment of foot infection in persons with diabetes (IWGDF 2019 update). Diabetes Metab Res Rev 36:e3280. doi:10.1002/dmrr.3280 32176444

[3] Prompers L, Huijberts M, Apelqvist J, Jude E, Piaggesi A, Bakker K, Edmonds M, Holstein P, Jirkovska A, Mauricio D, Ragnarson Tennvall G, Reike H, Spraul M, Uccioli L, Urbancic V, Van Acker K, van Baal J, van Merode F, Schaper N. 2007. High prevalence of ischaemia, infection and serious comorbidity in patients with diabetic foot disease in Europe. baseline results from the eurodiale study. Diabetologia 50:18–25. doi:10.1007/s00125-006-0491-1 17093942

[4] Armstrong DG, Boulton AJM, Bus SA. 2017. Diabetic foot ulcers and their recurrence. N Engl J Med 376:2367–2375. doi:10.1056/NEJMra1615439 28614678

[5] Loïez C, Wallet F, Pischedda P, Renaux E, Senneville E, Mehdi N, Courcol RJ. 2007. First case of osteomyelitis caused by “*Staphylococcus pettenkoferi*”. J Clin Microbiol 45:1069–1071. doi:10.1128/JCM.02328-06 PMC1829132 17202276

[6] Aragón-Sánchez J, Lázaro-Martínez JL, Hernández-Herrero MJ, Quintana-Marrero Y, Cabrera-Galván JJ. 2010. Clinical significance of the isolation of *Staphylococcus epidermidis* from bone biopsy in diabetic foot osteomyelitis. Diabet Foot Ankle 1:10. doi:10.3402/dfa.v1i0.5418 PMC3284277 22396808

[7] Kear S, Smith C, Mirmiran R, Hofinger D. 2016. *Staphylococcus lugdunensis*: a rare pathogen for osteomyelitis of the foot. J Foot Ankle Surg 55:255–259. doi:10.1053/j.jfas.2014.06.019 25154651

[8] Nguyen MTT, Ahern NR, Train MK. 2020. *Staphylococcus schleiferi* diabetic foot osteomyelitis and bacteraemia in an immunocompromised host. BMJ Case Rep 13:e238302. doi:10.1136/bcr-2020-238302 PMC7643485 33148559

[9] Ahmad-Mansour N, Plumet L, Huc-Brandt S, Magnan C, Yahiaoui-Martinez A, Kissa K, Pantel A, Lavigne J-P, Molle V. 2021. Investigating pathogenicity and virulence of *Staphylococcus pettenkoferi:* an emerging pathogen. Int J Mol Sci 22:13614. doi:10.3390/ijms222413614 PMC8704685 34948410

[10] Magnan C, Ahmad-Mansour N, Pouget C, Morsli M, Huc-Brandt S, Pantel A, Dunyach-Remy C, Sotto A, Molle V, Lavigne J-P. 2022. Phenotypic and genotypic virulence characterisation of *Staphylococcus pettenkoferi* strains isolated from human bloodstream and diabetic foot infections. Int J Mol Sci 23:15476. doi:10.3390/ijms232415476 PMC9778964 36555117

[11] Abi Najem CN, Magnan C, Plumet L, Ahmad-Mansour N, Pouget C, Morsli M, Pantel A, Kissa K, Sotto A, Lavigne J-P, Molle V. 2024. Deciphering pathogenicity and virulence of the first *Staphylococcus debuckii* isolate from diabetic foot osteomyelitis. Front Cell Infect Microbiol 14:1489280. doi:10.3389/fcimb.2024.1489280 PMC11685071 39742337

[12] Magnan C, Plumet L, Morsli M, Dunyach-Remy C, Pouget C, Sotto A, Molle V, Lavigne J-P. 2025. A narrative review of coagulase-negative staphylococci in diabetic foot infections. Clin Microbiol Rev 38:e00121-25. doi:10.1128/cmr.00121-25 PMC12697129 41186420

[13] Igimi S, Kawamura S, Takahashi E, Mitsuoka T. 1989. *Staphylococcus felis*, a new species from clinical specimens from cats. Int J Syst Bacteriol 39:373–377. doi:10.1099/00207713-39-4-373

[14] Kwaszewska A, Lisiecki P, Szemraj M, Szewczyk EM. 2015. Animal *Staphylococcus felis* with the potential to infect human skin. Med Dosw Mikrobiol 67:69–78. 26591658

[15] Sips GJ, van Dijk MAM, van Westreenen M, van der Graaf-van Bloois L, Duim B, Broens EM. 2023. Evidence of cat-to-human transmission of *Staphylococcus felis*. J Med Microbiol 72. doi:10.1099/jmm.0.001661 36763079

[16] Sotto A, Lina G, Richard J-L, Combescure C, Bourg G, Vidal L, Jourdan N, Etienne J, Lavigne J-P. 2008. Virulence potential of *Staphylococcus aureus* strains isolated from diabetic foot ulcers: a new paradigm. Diabetes Care 31:2318–2324. doi:10.2337/dc08-1010 PMC2584189 18809632

[17] Plumet L, Morsli M, Ahmad-Mansour N, Clavijo-Coppens F, Berry L, Sotto A, Lavigne J-P, Costechareyre D, Molle V. 2023. Isolation and characterization of new bacteriophages against staphylococcal clinical isolates from diabetic foot ulcers. Viruses 15:2287. doi:10.3390/v15122287 PMC10747802 38140529

[18] Kim H, Lee H, Shin D. 2012. The FeoA protein is necessary for the FeoB transporter to import ferrous iron. Biochem Biophys Res Commun 423:733–738. doi:10.1016/j.bbrc.2012.06.027 22705302

[19] Shin M, Jin Y, Park J, Mun D, Kim SR, Payne SM, Kim KH, Kim Y. 2021. Characterization of an antibacterial agent targeting ferrous iron transport protein FeoB against *Staphylococcus aureus* and gram-positive bacteria. ACS Chem Biol 16:136–149. doi:10.1021/acschembio.0c00842 33378170

[20] Becker K, Heilmann C, Peters G. 2014. Coagulase-negative staphylococci. Clin Microbiol Rev 27:870–926. doi:10.1128/CMR.00109-13 PMC4187637 25278577

[21] Otto M. 2009. *Staphylococcus* epidermidis--the “accidental” pathogen. Nat Rev Microbiol 7:555–567. doi:10.1038/nrmicro2182 PMC2807625 19609257

[22] Flannagan RS, Brozyna JR, Kumar B, Adolf LA, Power JJ, Heilbronner S, Heinrichs DE. 2022. *In vivo* growth of *Staphylococcus lugdunensis* is facilitated by the concerted function of heme and non-heme iron acquisition mechanisms. J Biol Chem 298:101823. doi:10.1016/j.jbc.2022.101823 PMC9052147 35283192

[23] Tock MR, Dryden DTF. 2005. The biology of restriction and anti-restriction. Curr Opin Microbiol 8:466–472. doi:10.1016/j.mib.2005.06.003 15979932

[24] Oliveira PH, Touchon M, Rocha EPC. 2014. The interplay of restriction-modification systems with mobile genetic elements and their prokaryotic hosts. Nucleic Acids Res 42:10618–10631. doi:10.1093/nar/gku734 PMC4176335 25120263

[25] Delaune A, Poupel O, Mallet A, Coic Y-M, Msadek T, Dubrac S. 2011. Peptidoglycan crosslinking relaxation plays an important role in *Staphylococcus aureus* WalKR-dependent cell viability. PLoS One 6:e17054. doi:10.1371/journal.pone.0017054 PMC3046168 21386961

