# Supplementary material for: First characterization of Staphylococcus felis in diabetic foot osteomyelitis: from intracellular persistence to phage treatment
Source: Microbiol Spectr. 2026 Sep 14;14(10):e02340-25. doi: 10.1128/spectrum.02340-25 (PMC13689204; doi:10.1128/spectrum.02340-25)
Supplement: Supplementary file 1 [file spectrum.02340-25-s0001.pdf]

**Table S1. Resistome analysis of *S. felis* NSF001, NSF002, ATCC 49168, and *S. aureus* NSA739 strains** (accession numbers provided).

| Gene | Annotation | NSF001 | NSF002 | ATCC 49168 | NSA739 |
|---|---|---|---|---|---|
| *sdrM* | multidrug efflux MFS transporter SdrM | WP_001237506.1 | WP_001237506.1 | WP_001237506.1 | WP_001237506.1 |
| *norA* | multidrug efflux MFS transporter NorA | WP_001041274.1 | WP_001041274.1 | WP_001041274.1 | - |
| *norB* | multidrug efflux MFS transporter NorB | - | - | - | WP_000414695.1 |
| *norC* | multidrug efflux MFS transporter NorC | - | - | - | WP_001006441.1 |
| *sepA* | SepA family multidrug efflux transporter | WP_001829729.1 | WP_001829729.1 | WP_001829729.1 | WP_000636857.1 |
| *tet(38)* | tetracycline efflux MFS transporter Tet(38) | WP_001100299.1 | WP_001100299.1 | WP_001100299.1 | - |
| *mecA* | PBP2a family beta-lactam-resistant peptidoglycan transpeptidase MecA | - | - | - | WP_001801873.1 |
| *catA* | type A-8 chloramphenicol O-acetyltransferase | - | - | - | WP_000143686.1 |
| - | lantibiotic immunity ABC transporter MutE/EpiE family permease subunit | WP_009888312.1 | WP_009888312.1 | - | - |
| - | lantibiotic protection ABC transporter ATP-binding protein | WP_011986262.1 | WP_011986262.1 | - | - |
| - | ABC-F type ribosomal protection protein | WP_000150916.1<br>WP_000602057.1 | WP_000150916.1<br>WP_000602057.1 | - | - |

**Table S2. Comparison of virulence-related genes between *S. felis* NSF001, NSF002, ATCC 49168, and *S. aureus* NSA739 strains** (Accession or UniRef50 numbers provided)

| | | Putative Genetic Determinants of virulence | NSF001 | NSF002 | ATCC 49168 | NSA739 |
|---|---|---|---|---|---|---|
| **Toxins** | - | hemolysin III family protein | WP_000047255.1 | WP_000047255.1 | WP_000047255.1 | WP_000047255.1 |
| | *hlgA, hlgB, hlgC* | bi-component gamma-hemolysin HlgAB subunit A ; HlgCB subunit C ; HlgAB/HlgCB subunit B | - | - | - | WP_000594519.1 WP_000916704.1 WP_000783428.1 |
| | *hyl* | alpha-hemolysin | - | - | - | WP_000857488.1 |
| | *sel26, sea, selX* | staphylococcal enterotoxin type 26, type A, -like toxin X | - | - | - | WP_000889280.1 WP_000750412.1 WP_000475325.1 |
| | *etD* | Putative exfoliative toxin D-like serine peptidase | P09332 | P09332 | P09332 | - |
| | *lukDE* | Leucotoxin LukE and LukD | - | - | - | WP_088621909 WP_088621910 |
| **Avoid Host Immune Response and Invasiveness** | *capA* | capsular polysaccharide type 5/8 biosynthesis protein CapA | - | - | - | WP_000446299.1 |
| | *cap8B* | type 8 capsular polysaccharide synthesis protein Cap8B | - | - | - | WP_000037332.1 |
| | *cap8C* | type 8 capsular polysaccharide synthesis protein Cap8C | - | - | - | WP_000565304.1 |
| | *cap8D* | type 8 capsular polysaccharide synthesis protein Cap8D | - | - | - | WP_000940790.1 |
| | *cap8E* | type 8 capsular polysaccharide synthesis protein Cap8E | - | - | - | WP_000459062.1 |
| | *cap8F* | type 8 capsular polysaccharide synthesis protein Cap8F | - | - | - | WP_001028283.1 |
| | *cap8G* | type 8 capsular polysaccharide synthesis protein Cap8G | - | - | - | WP_000413171.1 |
| | *cap8L* | type 8 capsular polysaccharide synthesis protein Cap8L | - | - | - | WP_001291020.1 |
| | *cap8M* | type 8 capsular polysaccharide synthesis protein Cap8M | - | - | - | WP_000825098.1 |

| | | | | | |
|---|---|---|---|---|---|
| *capN* | capsular polysaccharide type 5/8 biosynthesis epimerase CapN | - | - | - | WP_001230679.1 |
| *cap8O* | type 8 capsular polysaccharide synthesis protein Cap8O | WP_000779504.1 | WP_000779504.1 | WP_000779504.1 | WP_000779504.1 |
| *cap8P* | type 8 capsular polysaccharide synthesis protein Cap8P | - | - | - | WP_000723436.1 |
| *sbi* | Immunoglobulin-binding protein Sbi | WP_000792559.1 | WP_000792559.1 | - | WP_000792559.1 |
| *lip2* | YSIRK domain-containing triacylglycerol lipase Lip2/Geh | WP_000943819.1 | WP_000943819.1 | - | WP_000943819.1 |
| *lip1* | YSIRK domain-containing triacylglycerol lipase Lip1 | - | - | - | WP_000842040.1 |
| *lipA* | Lipoyl synthase | WP_000201875.1 | WP_000201875.1 | WP_000201875.1 | WP_000201875.1 |
| *eno* | Enolase | WP_001829595.1 | WP_001829595.1 | WP_001829595.1 | WP_001121760.1 |
| *katA* | Catalase | WP_000082539.1 | WP_000082539.1 | WP_000082539.1 | WP_000082539.1 |
| *tuf* | Elongation factor tu | WP_001832289.1 | WP_001832289.1 | WP_001832289.1 | WP_001040568.1 |
| *lspA* | Lipoprotein signal peptidase | WP_000549207.1 | WP_000549207.1 | WP_000549207.1 | WP_000549207.1 |
| *lgt* | Lipoprotein diacylglycerol transferase | WP_000513305.1 | WP_000513305.1 | WP_000513305.1 | WP_000513305.1 |
| *scn* | Complement inhibitor SCIN-A | WP_000702262.1 | WP_000702262.1 | WP_000702262.1 | WP_000702263.1 |
| *coA* | Coagulase | - | - | - | WP_000744096.1 |
| *spA* | Staphylococcal protein A | - | - | - | WP_000728765.1 |
| *vwb* | von Willebrand factor binding protein Vwb | - | - | - | WP_000791702.1 |
| *gehC* | YSIRK domain-containing triacylglycerol lipase GehC | WP_002437845.1 | WP_002437845.1 | WP_002437845.1 | - |
| *isaA* / *isaB* | lytic transglycosylase IsaA/immunodominant staphylococcal antigen IsaB | - | - | - | WP_000751263.1 WP_001044560.1 |
| *sak* | Staphylokinase | - | - | - | WP_000920038.1 |
| *foeA* | Ferrous iron transport protein A | _ | A0A0A8HRQ5 | A0A0A8HRQ5 | - |
| *foeB* | Ferrous iron transport protein B | A0A7Z8E306 | A0A7Z8E306 | A0A7Z8E306 | A0A7Z8E306 |

| | | | | | | |
|---|---|---|---|---|---|---|
| **Protease activity or implicated in proteolysis** | *clpP* | ATP-dependent Clp endopeptidase proteolytic subunit ClpP | WP_001049165.1 | WP_001049165.1 | WP_001049165.1 | WP_001049165.1<br>WP_225426975.1<br>WP_000642728.1 |
| | *clpB* | ATP-dependent chaperone ClpB | WP_001829334.1 | WP_001829334.1 | WP_001829334.1 | WP_000353954.1 |
| | *clpX* | ATP-dependent Clp protease ATP-binding subunit ClpX | WP_001830765.1 | WP_001830765.1 | WP_001830765.1 | WP_000472302.1 |
| | *clpL* | ATP-dependent Clp protease ATP-binding subunit ClpL | - | - | - | WP_001058993.1 |
| | *aur* | zinc metalloproteinase aureolysin | - | - | - | WP_001821522.1 |
| **Regulation** | *nsaS/nsaR* | Sensor histidine kinase/DNA-binding response regulator | - | - | - | WP_000143425.1<br>WP_000697877.1 |
| | *srrA/srrB* | Transcriptional regulatory protein SrrA / Sensor protein SrrB | WP_000987774.1<br>WP_001831066.1 | WP_000987774.1<br>WP_001831066.1 | WP_000987774.1<br>WP_001831066.1 | WP_000987774.1<br>WP_000064078.1 |
| | *vraSR* | Sensor Pt VRAS Response regulator Pt VRAR | WP_001017131.1<br>WP_000153535.1 | WP_001017131.1<br>WP_000153535.1 | WP_001017131.1<br>WP_000153535.1 | WP_001017131.1<br>WP_000153535.1 |
| | *saeSR* | Histidine protein kinase SaeS /Response regulator SaeR | WP_000244415.1<br>WP_000149344.1 | WP_000244415.1<br>WP_000149344.1 | WP_000244415.1<br>WP_000149344.1 | WP_000244415.1<br>WP_000149344.1 |
| **Other** | *cspA* | cold shock protein CspA | WP_001831260.1 | WP_001831260.1 | WP_001831260.1 | WP_000809131.1 |
| | *scpA* | cysteine protease staphopain A | - | - | - | WP_000827746.1 |
| | *comGA*<br>*comGB*<br>*comGC*<br>*comGD* | competence type IV pilus ATPase ComGA<br>competence type IV pilus assembly protein ComGB<br>ccompetence type IV pilus major pilin ComGC<br>competence type IV pilus minor pilin ComGD | WP_001831109.1<br>WP_001831131.1<br>WP_000472256.1<br>WP_001788899.1 | WP_001831109.1<br>WP_001831131.1<br>WP_000472256.1<br>WP_001788899.1 | WP_001831109.1<br>WP_001831131.1<br>WP_000472256.1<br>WP_001788899.1 | WP_000697220.1<br>WP_000776422.1<br>WP_000472256.1<br>WP_001788899.1 |
| | *comGF* | competence type IV pilus minor pilin ComGF | - | - | - | WP_001788897.1 |

| | | | | | |
|---|---|---|---|---|---|
| - | competence protein ComK | WP_001829272.1 | WP_001829272.1 | WP_001829272.1 | WP_000287265.1 |
| - | competence protein CoiA family protein | WP_001829282.1 | WP_001829282.1 | WP_001829282.1 | WP_000959282.1 |
| - | DNA internalization-related competence protein ComEC/Rec2 | - | - | - | WP_000953300.1 |
| *esxA* | WXG100 family type VII secretion effector EsxA | - | - | - | WP_001240826.1 |
| *esaA* | type VII secretion protein EsaA | - | - | - | WP_000728958.1 |
| *essB* | type VII secretion protein EssB | - | - | - | WP_000240338.1 |
| *essC* | type VII secretion protein EssC | - | - | - | WP_000549287.1 |
| *esaC* | type VII secretion substrate EsaC | - | - | - | WP_001010292.1 |
| *esxB* | WXG100 family type VII secretion effector EsxB | - | - | - | WP_000509671.1 |
| *esxD* | type VII secretion system extracellular protein EsxD | - | - | - | WP_000175722.1 |
| *nikE* | nickel import ATP-binding protein NikE | WP_000173690.1 | WP_000173690.1 | - | - |

**Table S3. Comparison of biofilm-related genes between *S. felis* NSF001, NSF002, ATCC 49168, and *S. aureus* NSA739 strains** (Accession or UniRef50 numbers provided)

| Biofilm steps | | Putative Genetic Determinants of biofilm production | | NSF001 | NSF002 | ATCC 49168 | NSA739 |
|---|---|---|---|---|---|---|---|
| INITIAL ADHESION | to Abiotic Surfaces | *bhp* | Biofilm associated protein | - | - | - | - |
| | | *sasC, sasF* | Surface Proteins, cell-wall-anchored protein SasF | - | - | - | WP_001050566.1<br>WP_001151890.1 |
| | | *sasA* | serine-rich repeat glycoprotein adhesin SasA | - | - | - | - |
| | | *ebpS* | elastin-binding protein EbpS | WP_107515759.1 | WP_107515759.1 | WP_107515759.1 | WP_000069289.1 |
| | | *eap* | extracellular adherence protein Eap/Map | - | - | - | WP_001557458.1 |
| | | *emp* | extracellular matrix protein-binding adhesin Emp | - | - | - | WP_000728068.1 |
| | to Biotic Surfaces | *fnbpA* | Fibronectin binding protein A | - | - | - | WP_000794614.1 |
| | | *fnbpB* | Fibronectin binding protein B | WP_000841404.1 | WP_000841404.1 | WP_000841404.1 | WP_000841404.1 |
| | | AtlE | Autolysin E | - | - | - | W8UVI1 |
| | | *fbl, fib* | Fibrinogen binding protein | - | - | - | - |
| | | *clfA, clfB* | Clumping factors A and B | - | - | - | WP_001056195.1<br>WP_000745891.1 |
| | | *vwb* | von Willebrand factor binding protein Vwb | - | - | - | WP_000791702.1 |
| | | *sdrC, sdrD, sdrE* | MSCRAMM family adhesin SdrC, SdrD, SdrE | - | - | - | WP_001060462.1<br>WP_000934467.1<br>WP_000610259.1 |

| | | | | | | | |
|---|---|---|---|---|---|---|---|
| MATURATION | Intercellular Aggregation | *icaADBC* | Polysaccharide Intercellular Adhesion (PIA) | - | - | - | WP_001159430.1<br>WP_000240580.1<br>WP_000877369.1<br>WP_000723836.1 |
| | | *isdA, isdB* | Iron-regulated surface determinant protein A, B | - | - | - | WP_000160859.1<br>WP_001041583.1 |
| | | *sasG* | *S. aureus* surface protein G | - | - | - | - |
| | | *sbp* | *S. epidermidis* biofilm accumulation | - | - | - | - |
| | Biofilm Structuring | *splB,splC, splD, splF* | Serine proteases SplB,SplC,SplD, SplF | - | - | - | WP_001039454.1<br>WP_001038872.1<br>WP_001038704.1<br>WP_001038752.1<br>WP_001038752.1 |
| | | *splA* | Serine protease SplA | WP_001039427.1 | WP_001039427.1 | WP_001039427.1 | WP_001039427.1 |
| REGULATION | | *icaR* | Biofilm operon icaADBC regulator | - | - | - | WP_000653261.1 |
| | | *agrACDB* | Accessory gene regulators A,C, D and B | Q5HMY2<br>S2YFB1<br>A0A3A0VKE5<br>P61649 | Q5HMY2<br>S2YFB1<br>A0A3A0VKE5<br>P61649 | Q5HMY2<br>S2YFB1<br>A0A3A0VKE5<br>P61649 | Q5HMY2<br>O07911<br>Q53643<br>Q6GF36 |
| | | *sigB* | RNA polymerase sigma factor SigB | WP_002440602.1 | WP_002440602.1 | WP_002440602.1 | WP_001041111.1 |
| | | *rsbUVW* | / | A0A0D6XSI7<br>P60070<br>Q8Y8K6 | A0A0D6XSI7<br>P60070<br>Q8Y8K6 | A0A0D6XSI7<br>P60070<br>Q8Y8K6 | A0A0D6XSI7<br>P60070<br>Q8Y8K6 |
| | | *lytSR* | LytSR two-component regulatory system | WP_000925394.1<br>WP_000645452.1 | WP_000925394.1<br>WP_000645452.1 | WP_000925394.1<br>WP_000645452.1 | WP_000925394.1<br>WP_000645452.1 |

| | | | | | | |
|---|---|---|---|---|---|---|
| | *arlRS* | ArlRS two-component system | Q99U73; Q49XM6 | Q99U73; Q49XM6 | Q99U73; Q49XM6 | WP_000192137.1 WP_000166801.1 |
| | *mgrA* | HTH-type transcriptional regulator MgrA | WP_001283444.1 | WP_001283444.1 | WP_001283444.1 | WP_001283444.1 |
| | *cidA* | Holin-like protein CidA | WP_000549734.1 | WP_000549734.1 | WP_000549734.1 | WP_000549734.1 |
| | *lrgA* | Antiholin-like protein LrgA | WP_001792906.1 | WP_001792906.1 | WP_001792906.1 | WP_001792906.1 |
| | *sarA* | Transcriptional regulator SarA | WP_001830809.1 | WP_001830809.1 | WP_001830809.1 | WP_001018677.1 |
| **DISPERSION** | *pmtA* | phenol-soluble modulin export ABC transporter ATP-binding protein PmtA | WP_001830432.1 | WP_001830432.1 | WP_001830432.1 | WP_000991306.1 |
| | *pmtB* | phenol-soluble modulin export ABC transporter permease subunit PmtB | WP_002494112.1 | WP_002494112.1 | WP_002494112.1 | WP_000645727.1 |
| | *pmtC* | phenol-soluble modulin export ABC transporter ATP-binding protein PmtC | WP_001830444.1 | WP_001830444.1 | WP_001830444.1 | WP_000763043.1 |
| | *pmtD* | phenol-soluble modulin export ABC transporter permease subunit PmtD | WP_001830379.1 | WP_001830379.1 | WP_001830379.1 | WP_001221651.1 |

**Table S4. Genomic variants identified between *Staphylococcus felis* NSF001 and NSF002 using** SNIPPY analysis with functional annotation performed via BAKTA.

| Contig | Genomic Position | Variant Type | NSF001 Allele | NSF002 Allele | Feature Type | Nucleotide Change (CDS Position) | Amino Acid Change (Protein Position) | Variant Effect | Product Annotation |
|---|---|---|---|---|---|---|---|---|---|
| 1 | 2240 | snp | T | C | CDS | 316/561 | 106/186 | missense_variant c.316A>G p.Asn106Asp | 16S rRNA (Cytosine(1402)-N(4))-methyltransferase |
| 1 | 80995 | snp | G | A | CDS | 994/1701 | 332/566 | missense_variant c.994G>A p.Val332Ile | Sensor protein kinase WalK |
| 1 | 241681 | snp | A | G | CDS | 447/453 | 149/150 | missense_variant c.447A>G p.Ile149Met | transcriptional regulator ArgR |
| 1 | 322444 | snp | C | T | CDS | 425/501 | 142/166 | missense_variant c.425C>T p.Thr142Ile | PTS glucose transporter subunit IIA |
| 1 | 522610 | snp | T | C | CDS | 523/744 | 175/247 | missense_variant c.523A>G p.Thr175Ala | Stp1/IreP family PP2C-type Ser/Thr phosphatase |
| 2 | 68422 | snp | G | A | CDS | 7/954 | 3/317 | stop_gained c.7C>T p.Gln3* | Ring-cleaving dioxygenase |
| 2 | 72289 | snp | T | C | | | | | |
| 2 | 124488 | ins | G | GA | CDS | 2286/2790 | 762/929 | frameshift_variant c.2285dupA p.Asn762fs | Type I restriction enzyme endonuclease subunit |
| 2 | 127749 | snp | A | C | CDS | 528/1233 | 176/410 | missense_variant c.528A>C p.Leu176Phe | restriction endonuclease subunit S |
| 5 | 82461 | snp | T | G | CDS | 200/1422 | 67/473 | missense_variant c.200A>C p.Glu67Ala | amidophosphoribosyltransferase |
| 5 | 86814 | snp | G | T | CDS | 773/1125 | 258/374 | missense_variant c.773C>A p.Ala258Asp | 5-(carboxyamino)imidazole ribonucleotide synthase |
| 5 | 138382 | del | AT | A | CDS | 188/840 | 63/279 | frameshift_variant c.188delA p.Asn63fs | hypothetical protein |
| 6 | 84185 | snp | G | T | CDS | 149/675 | 50/224 | missense_variant c.149C>A p.Ala50Glu | Putative N-acetylmannosamine-6-phosphate 2-epimerase |
| 7 | 80031 | snp | G | A | CDS | 172/1827 | 58/608 | missense_variant c.172C>T p.Leu58Phe | cell wall metabolism sensor histidine kinase WalK |
| 9 | 15816 | snp | A | C | CDS | 35/297 | 12/98 | missense_variant c.35A>C p.Gln12Pro | 30S ribosomal protein S6 |
| 9 | 36262 | ins | G | GT | CDS | 222/261 | 74/86 | frameshift_variant c.221dupT p.His75fs | Hypoxanthine-guanine phosphoribosyltransferase |
| 13 | 11845 | snp | T | G | | | | | |
| 21 | 1754 | del | CA | C | CDS | 12/519 | 4/172 | frameshift_variant c.12delA p.Val5fs | Type 1 glutamine amidotransferase |
| 23 | 9828 | snp | G | A | CDS | 131/678 | 44/225 | missense_variant c.131C>T p.Ala44Val | DUF4887 domain-containing protein |
| 26 | 10836 | snp | T | C | CDS | 1979/2139 | 660/712 | missense_variant c.1979A>G p.Gln660Arg | hypothetical protein |

| | | | | | | | | | |
|---|---|---|---|---|---|---|---|---|---|
| 26 | 10848 | snp | G | A | CDS | 1967/2139 | 656/712 | missense_variant c.1967C>T p.Pro656Leu | hypothetical protein |
| 26 | 11991 | snp | G | A | CDS | 824/2139 | 275/712 | missense_variant c.824C>T p.Thr275Met | hypothetical protein |
| 26 | 12651 | snp | T | G | CDS | 164/2139 | 55/712 | missense_variant c.164A>C p.Lys55Thr | hypothetical protein |
| 26 | 12720 | snp | A | T | CDS | 95/2139 | 32/712 | missense_variant c.95T>A p.Leu32Gln | hypothetical protein |
